# Immediate impacts of soybean cover crop on bacterial community composition and diversity in soil under long-term *Saccharum* monoculture

**DOI:** 10.7717/peerj.15754

**Published:** 2023-08-22

**Authors:** Himaya Mula-Michel, Paul White, Anna Hale

**Affiliations:** 1 DOE Center for Advanced Bioenergy and Bioproducts Innovation, USDA-ARS, Sugarcane Research Unit, Houma, LA, USA; 2 USDA-ARS, Sugarcane Research Unit, Houma, LA, USA

**Keywords:** Biodiversity, Mono-culture, Sugarcane, Cover crop, Conservation, Yield decline, Bacterial diversity, Community composition, Soybean, Sustainability

## Abstract

*Saccharum* yield decline results from long-term monoculture practices. Changes in cropping management can improve soil health and productivity. Below-ground bacterial community diversity and composition across soybean (*Glycine max* (L.) Merr) cover crop, Saccharum monoculture (30+ year) and fallowed soil were determined. Near full length (~1,400 base pairs) of 16S rRNA gene sequences were extracted from the rhizospheres of sugarcane and soybean and fallowed soil were compared. Higher soil bacterial diversity was observed in the soybean cover crop than sugarcane monoculture across all measured indices (observed operationational taxonomic units, Chao1, Shannon, reciprocal Simpson and Jackknife). Acidocateria, Proteobacteria, Bacteroidetes and Planctomycetes were the most abundant bacterial phyla across the treatments. Indicator species analysis identified nine indicator phyla. Planctomycetes, Armatimonadetes and candidate phylum FBP were associated with soybean; Proteobacteria and Firmicutes were linked with sugarcane and Gemmatimonadetes, Nitrospirae, Rokubacteria and unclassified bacteria were associated with fallowed soil. Non-metric multidimensional scaling analysis showed distinct groupings of bacterial operational taxonomic units (97% identity) according to management system (soybean, sugarcane or fallow) indicating compositional differences among treatments. This is confirmed by the results of the multi-response permutation procedures (A = 0.541, *p* = 0.00045716). No correlation between soil parameters and bacterial community structure was observed according to Mantel test (r = 211865, *p* = 0.14). Use of soybean cover-crop fostered bacterial diversity and altered community structure. This indicates cover crops could have a restorative effect and potentially promote sustainability in long-term *Saccharum* production systems.

## Introduction

Sugarcane (a complex hybrid of *Saccharum* spp.) is a tropical perennial plant cultivated worldwide accounting for 80% of global sugar production (ISO, 2016). In Louisiana, USA, the crop is vital to the state’s economy with an annual overall economic impact of $3 billion (American Sugarcane League, 2019). It is extensively cultivated as a monoculture with only a 6–7 month fallow period between 5-year sugarcane cycles. This long-term monoculture of sugarcane may lead to poor soil conditions that contribute to sugarcane yield decline (Pankhurst et al., 2003a, 2003b). Sugarcane yield decline is described as the loss of productive capacity of sugarcane varieties resulting from soil degradation under long-term sugarcane monoculture (Magarey et al., 1997). Such phenomenon has been observed in sugarcane fields across the globe such as in Australia (Magarey, 1996), Mauritius (Seeruttun et al., 2014), and Ethiopia (Dengia & Lantinga, 2016). Sugarcane yield decline is associated with degraded soils caused not only by the cultivation of sugarcane as monoculture, but also intensive tillage and use of heavy harvesting machinery (Pankhurst et al., 2005). The unintended consequences of long-term sugarcane monoculture systems include decrease in physical, chemical and biological attributes of the soil and proliferation of deleterious soil microorganisms which result to the decline in sugarcane productivity (Magarey, 1996; Garside et al., 1997; Stirling et al., 1999; Pankhurst et al., 2003a, 2005).

Energycanes, high-fiber sugarcane cultivars, are sometimes developed as part of sugarcane breeding programs (Knoll et al., 2021). Both energycane and sugarcane are derived from interspecific hybridizations among species of the *Saccharum* complex, with the most distinguishing characteristic being their end use. Frequently, energycane cultivars contain a higher fiber content than sugarcane, but the best energycanes are the *Saccharum* cultivars that best fit the desired processing platform. Taking into account that sugarcane and energycane are the same genus, and differ only by differentially enhanced desired traits through breeding, their physiological responses to environmental influences are similar. Since commercial production of energycane is in its infancy, when long-term experimental data is needed, the best model crop is sugarcane. Hence, in this study, sugarcane is used as the test crop to find whether introduction of a cover crop to long-term monoculture system has impact on below-ground microbiome. The productivity and health of agricultural systems depend greatly upon the functional processes carried out by soil microbial communities (Pankhurst et al., 1996). Microbial diversity promotes sustainability, as species richness ensures continuity of functions in the face of perturbations (Giller et al., 1997; Yachi & Loreau, 1999). Agricultural intensification can lead to a decline in biodiversity across the taxonomic spectrum. Degens et al. (2001) reported reduced catabolic evenness in cultivated soil compared to pasture (each >30 y). Resulting changes in soil chemical and physical properties including pH, soil moisture, and electrical conductivity led to reduced stress tolerance capacity of the soil. They concluded that as land-use alters soil properties, it also decreases the catabolic diversity of soil microorganisms, thus weakening the soil’s stress tolerance. Breaking of monoculture practices by using cover crops can buffer or possibly improve the detrimental consequence of intensive, monoculture farming.

Cover crops (also known as catch crops) are plants mostly grown after a primary crop is harvested (Abdalla et al., 2019). Benefits of cover crops on promoting agricultural sustainability include, soil erosion control (Repullo-Ruibérriz de Torres et al., 2018); reduction of N leaching during fallow period (Thapa, Mirsky & Tully, 2018); increasing soil organic carbon (Jian et al., 2020); reduction of incidence of certain soil pathogens (Fageria, Baligar & Bailey, 2005), suppression of early-season weeds (Osipitan et al., 2018) and enhancement of arbuscular mycorrhizal proliferation (Higo et al., 2019). Major advantages of using legumes as cover crop are increased of N input through biological N fixation and high quality of organic matter released to the soil in term of C/N ratio (Stagnari et al., 2017). They can foster taxonomic and functional diversity among soil biota, particularly for degraded soils (Giller et al., 1997). Sturz, Christie & Matheson (1998) suggested legumes as rotational crops. They offer added benefits such as carryover of residual populations of endophytically competent bacteria that are able to promote plant growth and inhibit disease development on top of the residual biologically fixed-nitrogen and improved soil structure. In sugarcane production fields, Pankhurst et al. (2003a) reported that rotational crops reduced the populations of known detrimental soil biota and significantly increased yield of subsequent sugarcane crops. In some locations a single-legume based break crop was sufficient to capture both benefits. Thus, incorporation of soybean as cover crop is a promising alternative in efforts to counteract the detrimental effects of long-term monoculture and achieving sustainable productivity in saccharum production systems.

In Louisiana’s sugarcane rotation, there is a period of fallow every 5 years between the termination of old ratoons and the planting of the new sugarcane crop. This period lasts 6–7 months between early Spring to Summer (*e.g*., February to August). Fields are often kept bare during this fallow period using tillage and/or herbicides. The potential for soil erosion with this system is high. Soybean is planted as a short-season rotational crop during this period for profit and ground cover. However, more growers are planting spring cover crops to conserve soil resources, and soybean fits in this system when grown as a green-manure crop. Despite this widespread practice among sugarcane growers, knowledge on the impact of cover crops on the soil microbial community dynamics and diversity under intensively managed sugarcane is limited.

In this study, we compared the bacterial diversity between the rhizospheres of a soybean cover crop and sugarcane. We determined whether introduction of soybean cover crop in a soil subjected to long-term sugarcane monoculture will induce diversity and compositional differences on rhizospheric microbial communities. We hypothesized that if different types of plants (*e.g*., legumes *vs* grasses) fostered distinct rhizosphere bacterial communities, adding a soybean cover crop into the monoculture sugarcane system would result in the change in bacterial community composition and diversity.

## Materials and Methods

### Experimental design

The experimental plots were established at the USDA-ARS Sugarcane Research Unit (SRU) Ardoyne Farm in Schriever, LA (29°38′02″N 90°50′05″W). The soil is a Cancienne silt loam (Fine-silty, mixed, super active, non-acid, hyperthermic Fluvaquentic Epiaquepts). Sugarcane has been grown continuously on the test fields for at least 30 years. Following sugarcane harvest in the winter of 2016, one portion of the test field was disked to terminate the sugarcane ratoons in anticipation of replanting in the summer of 2017. During this fallow period, in May 2017, soybean (*Glycine max*) was planted on one half of the fallowed field as a cover crop while the remainder was kept bare. This field was adjacent to a sugarcane field that was harvested in 2016 and left to ratoon in 2017. Sugarcane was fertilized in April 2017 with urea ammonium nitrate (32% N) and muriate of potash (Helena Chemical, Thibodaux, LA, USA) at 135 N and 68 K kg ha^−1^, respectively. Fallow and soybean treatments were not fertilized in 2017.

**Figure 1:**
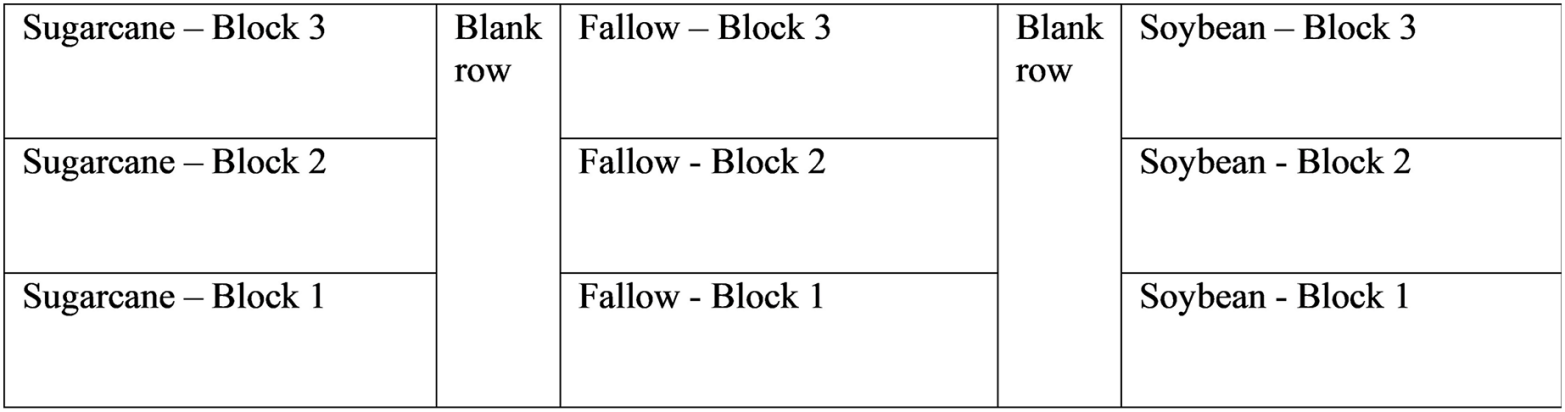
Experimental field layout showing treatments and replications.

The experimental layout was a randomized complete block design with three treatments with three replications each. Each treatment (soybean, sugarcane, or fallow) occupied 473 m^2^ and was divided lengthwise into 158 m^2^ blocks. Each block consists of two rows, spaced 1.83-m apart, separated by one blank row running lengthwise down field. Each block is 43.2-m in length × 3.66-m wide. A diagram of the experimental layout is shown in Fig. 1.

### Sample collection and processing

Soil samples were collected from the rhizospheres of soybean and sugarcane, and from the fallow soil, on July 21, 2017. Three randomly selected soybean and sugarcane plants were uprooted in each block to collect the rhizosphere samples. The loose soil was removed by shaking the plant roots over a basin. The remaining rhizospheric soil was manually removed using a scoopula, pooled and mixed thoroughly using a sieve to one composite sample. Three samples (0–15 cm depth) were collected from each block of the fallowed soil as well. The samples from each treatment replicate were mixed thoroughly using a sieve and pooled into a composite sample. Composite soil samples were stored at 4 °C until DNA extraction. The loose soil collected from the sugarcane and soybean root systems, and soil collected from fallow soil, were air-dried and ground for soil chemical analyses following standard analysis methods (Sparks et al., 1996).

### DNA extraction and sequencing

Genomic DNA was extracted using the DNeasy PowerSoil Isolation kit (Qiagen Cat. No. 12888-50) following the manufacturer’s protocol. Four separate extractions from each sample were pooled treatment-wise at the end of the extraction process. DNA samples were sent to MR DNA Laboratory in Shallowater, TX, USA for sequencing. High-throughput DNA sequencing was accomplished using MRDNA lab protocol (www.mrdnalab.com). Briefly, 16S rRNA genomic DNA were amplified using 27F (AGRGTTTGATCMTGGCTCAG) and 1492R (GGGTTACCTTGTTACGACTT) primers on the PacBio Sequel through bTEFA DNA analysis service. This primer set is a universal primer that amplifies the near full-length (~1,400 base pairs) of bacterial 16s rRNA gene. The 16S rRNA gene is conserved bacterial cells which contain hypervariable regions which can provide species-specific signature sequences. Thus, it is is widely used in identification of bacteria and phylogenetic studies.

PCR amplification was carried out in a single-step 30 cycle using HotStarTaq Plus Master Mix Kit (Qiagen, Valencia, CA, USA) under the following conditions: 94 °C for 3 min, followed by 28 cycles of 94 °C for 30 s; 53 °C for 40 s and 72 °C for 1 min; after which a final elongation step at 72 °C for 5 min was performed. All amplicons from different treatment samples were mixed in equal concentrations and purified using Ampure PB beads and sequenced using PacBio Sequel chemistry following the manufacturer’s protocols (Pacific Biosciences, Menlo Park, CA, USA). The library for each sample was prepared using SMRTbell Template Prep Kit (Pacific Biosciences, Menlo Park, CA, USA) following the manufacturer’s user guide. During library preparation, each sample underwent DNA damage and end repair as well as barcode adapter ligation. Subsequent to adapter ligation, each library pool went through Exonuclease III and VII digestion to remove the failed ligation products. After completion of initial DNA sequencing, each library underwent a secondary analysis, circular consensus sequencing (CCS), using PacBio’s CCS2 algorithm. The long read-length capability of the Sequel and the SMRT Bell library design allow for the polymerase to make multiple passes of the same template over the 10-h movie time. Each pass of the SMRT Bell template is known as a subread. The CCS2 algorithm aligns the subreads from each template to generate a consensus sequence thereby correcting the stochastic errors generated in the initial analysis. The CCS mean read length and read score were, 1,513 base pairs and 0.994, respectively.

### Sequence analysis

Raw sequences were initially processed, including chimera detection and removal, by the proprietary analysis pipeline (www.mrdnalab.com) of MR DNA. De-noised sequences were further analyzed using Mothur (V1.39.5) bioinformatics package (Schloss et al., 2009). Further screening and culling out of chimeric sequences were done using uchime (chimera.uchime) as implemented by Mothur (V1.39.5). Unique sequences with similarity scores above 80% were aligned against silva (silva.nr_v132.align) as reference and pre-clustered using default parameters to remove potentially erroneous amplicons. A Phylip-formatted distance matrix (dist.seqs; output = lt) was clustered into operational taxonomic units (OTU) (cluster.seqs) at 97% identity. Taxonomic classification of OTUs was done using silva.nr_v132.tax. Downstream analyses included make.shared, summary.single, and rarefaction.single commands. Sequences were submitted to GenBank (https://www.ncbi.nlm.nih.gov/genbank/) with accession number KDPR01000001–KDPR01063647.

The PC-Ord v7 software package (McCune & Mefford, 1999) was used for multivariate analyses using treatments x species matrix excluding the singletons. Difference in bacterial community composition was tested using Multi-response permutation procedures (MRPP) as implemented in PC-Ord (McCune & Mefford, 1999), using the Sorensen distance measure and rank transformation of the matrices. The A value in MRPP indicates a separation between cropping systems. When A = 0, groups are no more or less different than expected by chance while A = 1 means sample units within each group are identical (Peck, 2016). Graphical representation of community composition among sample units was done using nonmetric multidimensional scaling (NMS) using the Sorensen distance measure. NMS does not make distributional assumptions and is therefore suited for most ecological data (Peck, 2016). The “slow and thorough’ autopilot mode was used to determine the best ordination dimensionality using the Sorensen distance measure with 250 runs with real data and 500 iterations. A final two-dimension solution was selected with 3.7% stress and 0.00000 instability values. A Mantel test was performed to determine the relationship between an array of soil chemical properties and bacterial community composition. Indicator species analysis (ISA) was used to identify OTUs that were significantly associated with treatments. ISA evaluates the strength of the association between OTUs and treatments using the frequency and abundance of these OTUs in each treatment.

### Statistical analysis

An analysis of variance (ANOVA) was performed on soil properties and diversity indices using Proc Mixed was conducted using SAS version 9 software (SAS institute, Cary, NC, USA).

## Results

Soils from the treatment plots were slightly acidic (pH 5.97–6.0). No significant effect of the treatments on levels of SOM (1.74–9%), CEC (13–17.88 meq 100 g^−1^), NH_4_-N (1.67–2.6 ppm), P (17.67–25 ppm) and most of the micronutrients (Fe, 286–316 mg kg^−1^; Bo, 0.31–0.44 mg kg^−1^; Cu, 3.16–6.82 mg kg^−1^; Zn 1.92–3.77 mg kg^−1^; Al, 359–471 mg kg^−1^) were observed in this study. Nitrate-N (NO_3_-N, 0.4–3.8 ppm), K (58.33–118 mg kg^−1^), Ca (1,476–2,114 mg kg^−1^), Mg (339–413 mg kg^−1^), Na (20–33 mg kg^−1^), Mn (78–1,106 mg kg^−1^) and B (0.31–0.44 mg kg^−1^) were significantly higher in fallowed soil, compared to soils cultivated with sugarcane and soybean. Sulfur (S, 5.0–8.33 mg kg^−1^) and Co (0.57–0.81 mg kg^−1^) were comparable between sugarcane and the fallowed soil, but higher than the soybean-derived soil (Table 1).

**Table 1:** Mean values of the soil chemical properties (*n* = 3).

Soil properties	pH	SOM^†^ (%)	CEC^‡^ meq 100 g^−1^	NO_3_^-^-N	NH_4_^+^-N	P	K	S	Ca	Mg	Fe	Na	B	Mn	Cu	Zn	Al	Co
				ppm		mg kg^−1^												
Soybean	5.97	1.74	13.80	3.1ba	2.60	17.67	58.33c	5.00b	1476b	280.00b	286	23.67b	0.31b	78b	3.16	1.92	359	0.57b
Sugarcane	6.00	1.80	13.00	0.40b	1.67	19.00	80.33b	8.33a	1576ab	339.33b	309	20.00b	0.33b	98b	4.00	2.51	383	0.81a
Fallowed soil	6.00	1.9	17.88	3.80ab^§^	2.9	25.00	118.00a	7.33ab	2114a	413.00a	316	33.00a	0.44a	106a	6.82^ns^	3.77	471	0.78a
LSD (*p* < 0.05)	ns	ns	ns	*	ns	ns	*	*	*	*	ns	*	*	*	ns	ns	ns	*

**Notes:**

*Statistically significant at *p* < 0.05.

^†^SOM, soil organic matter.

^‡^CEC, cation exchange capacity.

^ns^indicates nonsignificant difference (*p* > 0.05).

^§^Different letters within a column indicate significant differences by LSD at the *p* < 0.05 level.

### Bacterial diversity

A total of 63,647 high-quality sequences of the near full length 16S rRNA gene were obtained after sequence quality filtering. A similarity level of 97% was used to cluster sequences into OTUs, representing species-level taxonomic classification (Schloss & Handelsman, 2005). There were 17,424 OTUs identified which were used to calculate the diversity indices.

**Figure 2:**
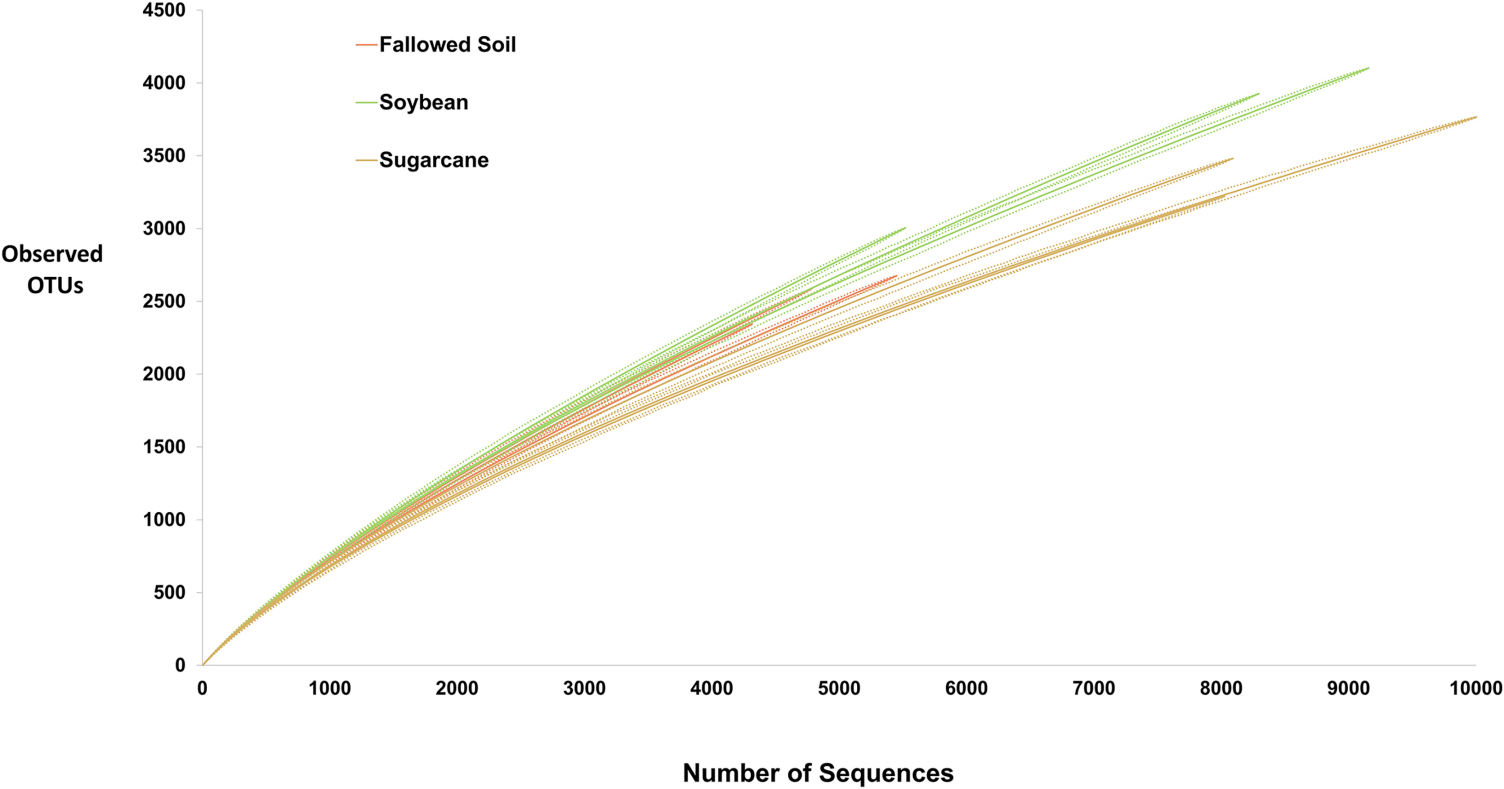
Rarefaction curves (solid lines) with 95% CI (dash lines) of the observed OTUs at 97% similarity for each treatment replicate.

High-throughput sequencing technology, such one used in this study, allowed discovery of species that were undetectable using culture-dependent techniques. Yet, exhaustive inventory of all species in microbial community remains impossible. Consequently, microbiologists must rely on samples to decipher actual diversity of microbial communities. Rarefaction curve is a common method used to assess taxon diversity in any system (Hughes et al., 2002) where number of observed OTUs are plotted against the number of sampled sequences. It measures OTUs observed with a given depth of sequencing, and are used to compare observed richness among communities that have been unequally sampled (Hughes & Hellmann, 2005). In this study, rarefaction curves for all treatment replicates did not approach the asymptote (Fig. 2) suggesting highly diversified bacterial communities in the samples and further sequencing would have generated more OTU’s. Soybean samples exhibited steepest curves suggesting highest bacterial richness among the treatments with sugarcane being the lowest. Similar trend was observed with other diversity indices presented in Table 2 where soybean had the highest value of all diversity measurements.

**Table 2:** Diversity indices as affected by treatments.

Management system	Chao1	Shannon	Reciprocal simpson (1/D)	Jackknife
Fallow	19,460 (4,352)ab	7.64 (0.12)b	939 (157)ab	24,086 (3,086)b
Soybean	26,922 (3,929)a	7.96 (0.04)a	1,054 (68)a	37,457 (4,530)a
Sugarcane	16,727 (9,058)b	7.586 (0.17)b	617 (234)b	28,872 (9,391)ab

**Note:**

The values represent the means while the values in parenthesis are the standard deviations (*n* = 3). OTU clustering was done at 97% similarity according to MOTHUR. Numbers with the same letters indicate not significant while numbers with different letters significant difference (*p* = 0.05).

For hyper-diverse assemblage with many undetected species, it is statistically impossible to obtain good estimate of species richness. Hence, Chao1 nonparametric richness estimator is often of more practical use than an imprecise point estimate (Chao & Chiu, 2016). It gives more weight to rare species (singletons and doubletons), therefore, useful for data sets with low-abundances species (Hughes & Hellmann, 2005) such as the data in this study. Chao1 predicted that only about 25–32% of the diversity were actually observed which is also depicted by failure of rarefaction curves to plateau (Fig. 2 and Table 2). Shannon index reflects richness and evenness; Simpson is weighted on dominance while Jackknife estimator is based on the presence or absence of a species. All of these diversity indicators were consistently highest in soybean-derived soil (Table 2).

### Community composition

**Figure 3:**
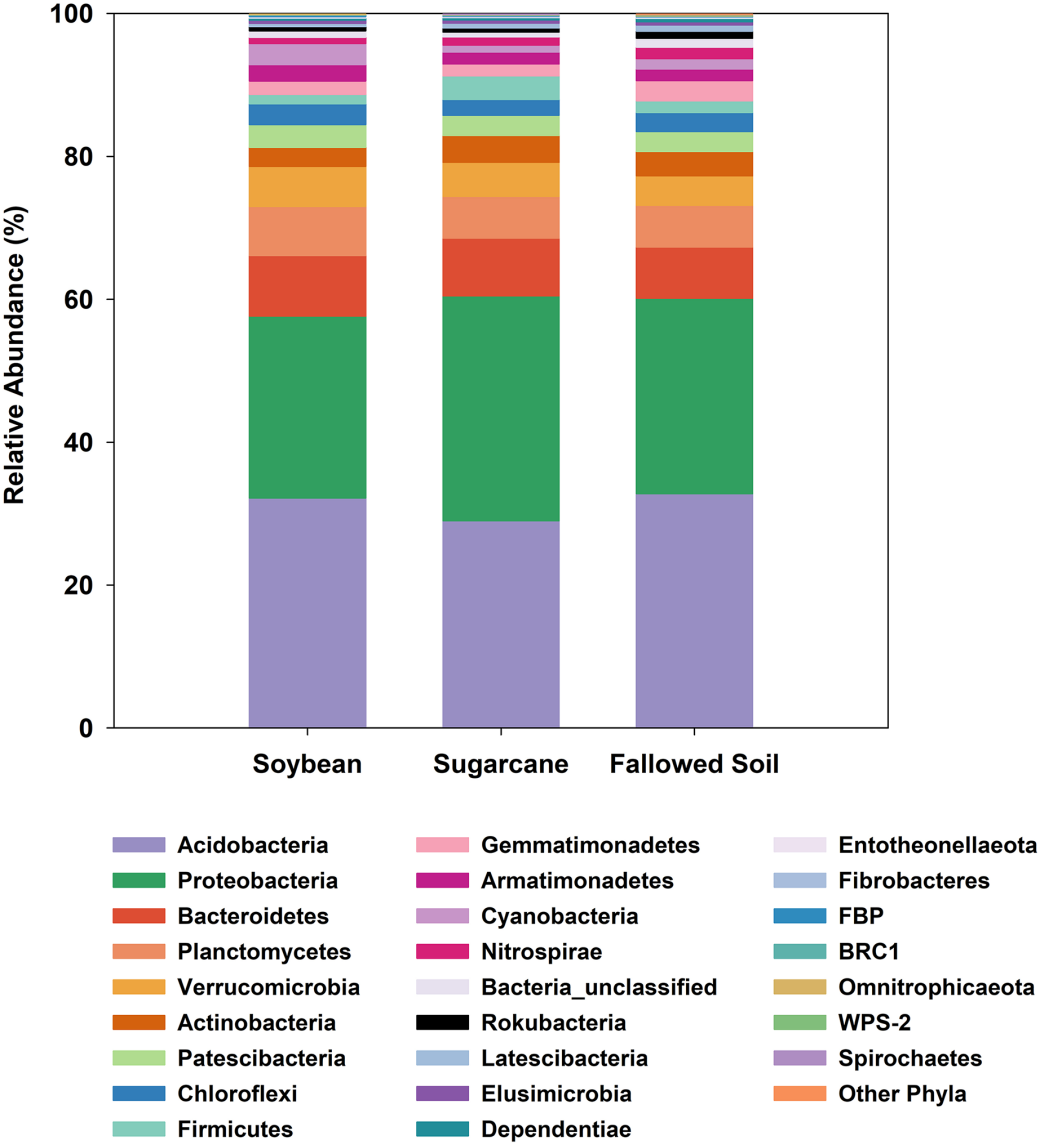
Relative abundances of bacterial phylotypes sequenced from DNA extracted from soybean and sugarcane rhizospheres, and fallowed soil. Each column represents the mean of three separate DNA extractions.

All three management systems were dominated by similar bacterial phyla of which Acidobacteria ranked the most dominant group comprising 29–33% followed by Proteobacteria (25–31%). The remaining phyla occurred at much lower abundances (<8%). Phyla with relative abundances of <0.2% were grouped together as “Other Phyla”. This included: WS2; Kiritimatiellaeota; FCPU426; Hydrogenedentes; WS4; Dadabacteria; Epsilonbacteraeota; Deinococcus-Thermus; GAL15 and Zixibacteria (Fig. 3). Indicator species analysis (ISA) was done on these phylotypes to determine which taxa had significant association with the treatments (*p* < 0.05). ISA identified nine indicator phyla. Planctomycetes, Armatimonadetes and candidate phylum FBP were associated with soybean; Proteobacteria and Firmicutes with sugarcane; and Gemmatimonadetes, Nitrospirae, Rokubacteria and unclassified bacteria with the fallowed soil (Table 3).

**Table 3:** Phylum-level taxa significantly associated with treatments determined by indicator species analysis (ISA).

Treatment	Taxa	Observed indicator values (IV)	IV from randomized groups		*p* value
			Mean (*n* = 3)	Standard deviation	
Soybean
	Planctomycetes	37.00	35.3	1.02	0.0246
	Armatimonadetes	40.90	36.3	1.78	0.0334
	FBP	59.3	44.0	6.22	0.0272
Sugarcane
	Proteobacteria	37.30	35.3	1.09	0.0472
	Firmicutes	52.60	43.1	4.66	0.0468
Fallowed soil
	Gemmatimonadetes	44.00	39.2	2.24	0.0184
	Nitrospirae	44.30	38	2.47	0.0338
	Rokubacteria	45.80	39.4	3.05	0.0338
	Unclassified bacteria	44.60	38.3	2.56	0.0286

**Figure 4:**
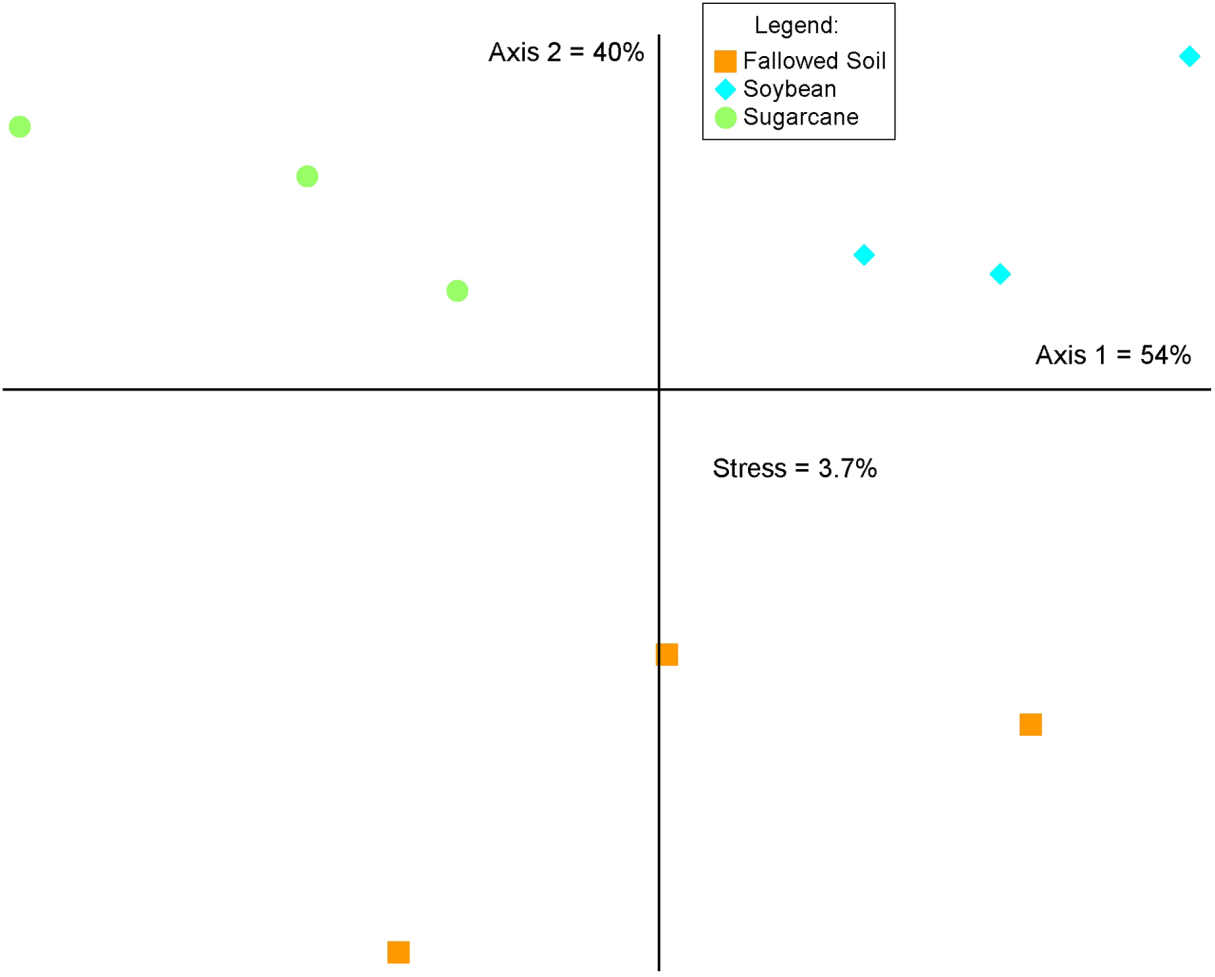
Non-metric multidimensional scaling (NMS) analysis of the relative abundances of the 16S rRNA genes (97% identity) across crop management practices. Percentages indicate the variance explained by each axis.

The three cropping systems differed in bacterial community composition as indicated in the MRPP test (A = 0.541, *p* = 0.00045716). This was corroborated by the graphical representation of the NMS ordination of which two axes accounted for 94% of the total variation of the species composition (Fig. 4). Bacterial communities grouped according to cropping systems. While each treatment were clustered separately, both soybean and sugarcane were distinctly separated from the fallowed soil. Mantel’s test on the soil chemical properties did not show significant relationship with bacterial community change and cropping systems (r = 0.211; *p* = 0.138).

## Discussion

This study evaluates the influence of soybean as a cover crop on bacterial community composition and diversity in a soil subjected to long-term sugarcane monoculture. Our results show higher bacterial diversity as indicated in all four measures of diversity in soybean compared to sugarcane and fallowed soil (Fig. 2 and Table 2) demonstrating the crop’s ability to recruit diverse bacterial communities. Inclusion of soybean as cover crop in long-term sugarcane monoculture triggers the proliferation of a variety of soil bacterial communities which can be attribute to the availability of new types of rhizodeposits (Schlatter et al., 2015; Vukicevich et al., 2016). Studies indicate that soybean rhizosphere recruits microbial communities with functional capabilities that increase plant nutrition (Mendes et al., 2014) and suppress disease incidence (Vukicevich et al., 2016). This information added to the favorability of soybean as cover crop in long-term sugarcane monoculture system.

The reduced microbial diversity in sugarcane monoculture soil observed in the current study is consistent with the low microbial diversity observed in other monoculture systems such as vetch (*Vicia sativa*) and oats *(Avena sativa L)* (Qiao et al., 2012); cotton (*Gossypium hirsutum****)*** (Acosta-Martínez et al., 2008) and soybean (Figuerola et al., 2015). Maintenance of viable diverse populations and functionality of microbial communities in the soil is essential to agricultural sustainability (Kennedy & Smith, 1995); including stability and resilience of soil under stress and disturbance (Griffiths et al., 2000; Torsvik & Øvreås, 2002). The low soil bacterial diversity in sugarcane monoculture is an indication of declining soil quality. Thus, the higher soil bacterial diversity in soybean cover crop implies the potential of the crop in restoring productivity in sugarcane monoculture system.

At a broader taxonomic (phylum) level of community characterization, we found that Acidobacteria and Proteobacteria comprised 59.4% of the total clones generated in this study, which were comparably distributed among the samples (Fig. 3). Both phyla dominate various soils and non-soil environments around the globe (Hugenholtz, Goebel & Pace, 1998; Janssen, 2006; Michel & Williams, 2011; Delgado-Baquerizo et al., 2018). Acidobacteria is a novel phylum whose members are very difficult to isolate and culture in the laboratory (Ward et al., 2009). However, it can comprise up to 50% of 16S rRNA clone libraries from soils (Quaiser et al., 2003; Michel & Williams, 2011). The ubiquity and abundance of Acidobacteria in soils can be attributed to their flexibility to metabolize wide range of carbon sources (Ward et al., 2009; Eichorst, Kuske & Schmidt, 2011). It is not surprising, then, that Acidobacteria is also dominant in our samples considering the ubiquitous nature of this phylotype. Proteobacteria is also a globally abundant phylum found in soils (Janssen, 2006; Elshahed et al., 2008; Delgado-Baquerizo et al., 2018) and rhizospheres (Philippot et al., 2013). It is comprised of highly diverse lineages with members performing key roles in ecological processes such as the carbon, nitrogen and sulfur cycling (Kersters et al., 2006). Proteobacteria were also reported to respond positively to the presence of vegetation and nutrient improvements (Smit et al., 2001; Thomson et al., 2010). The ratio between Proteobacteria and Acidobacteria may reflect the trophic status of the environments, with higher values reflecting copiotrophic systems (Smit et al., 2001; Hartman et al., 2008).

In this study, there is variability in the Proteobacteria and Acidobacteria ratios among the treatments (soybean = 0.79; sugarcane = 1.08; fallowed soil = 0.83). We speculate that continuous fertilizer inputs in monoculture sugarcane result in a higher supply of nutrients, thus supporting copiotrophic microbes such as the Proteobacteria. ISA analysis performed on these phylotypes identified nine phyla that are associated with treatment (Table 3). Association of these taxa to a particular treatment may be a response to the unique characteristics of such systems at the time of this study. For example, the copiotropic aspect of Proteobacteria (Bastida et al., 2015) may have found a better fit with sugarcane because of a sustained nutrient supply originating from either the continuous fertilizer inputs or the perennial crops’ carbohydrate reserve cycle. This observation is substantiated with the results of another study where a higher abundance of Proteobacteria was also observed in other mulberry and soybean monoculture systems (Li, Wu & Gao, 2018).

The higher NH_4_-N level in the fallowed soil (Table 1) as compared to the other two treatments may favor for the proliferation of Nitrospirae (Table 3). The OTUs under the Nitrospirae phyla belonged to the genus Nitrospira, an ecologically and phylogenetically diverse group, widely known for their nitrite-oxidizing capabilities (Koch et al., 2015). Recent studies also demonstrated that the most environmentally widespread clade of this diverse genus are able to perform complete ammonium oxidation (comammox Nitrospira) (Daims et al., 2015; Koch et al., 2015). It is interesting to note that the fallowed soil has significantly higher NO_3_-N where it is not fertilized and Nitrospirae is significantly associated with this treatment. It is plausible that higher NO_3_-N in fallowed soil could have been a product of the biogeochemical processes carried out by Nitrospirae or possibly by lack of plant uptake considering that this treatment is kept bare of vegetation.

Change in soil bacterial community structure with cropping system is visually demonstrated in the NMS ordination plot where bacterial communities are segregated according to treatments (Fig. 4). This variation is confirmed by the MRPP analysis (A = 0.541, *p* = 0.00045716) which shows significant differences in community composition among the cropping systems. This illustrates that a break in cultural practice from a long-term monoculture scheme drives changes in the underground bacterial microbiome. Similar observations in other studies were reported where the introduction of new plant species in soils with long-term agricultural histories (Maul & Drinkwater, 2010) and short fallows (Li, Wu & Gao, 2018) induced changes in microbial community diversity and composition. Soil properties and plant species are known as the main determinants in soil microbial community composition and structure in the rhizosphere (Berg & Smalla, 2009; Philippot et al., 2013) In this study, results of the Mantel test (r = 0.276; *p* = 0.138) show a non-significant correlation between the soil properties and bacterial community structure, indicating crop species exerted a considerable influence on the bacterial community composition.

Specificity of plant species to recruit distinct microbial populations is well documented (Kourtev, Ehrenfeld & Häggblom, 2002; Costa et al., 2006; Haichar et al., 2008; Berg & Smalla, 2009) with the process driven by root exudation and deposition (Jaeger et al., 1999; Kourtev, Ehrenfeld & Häggblom, 2002). Results of our study are in agreement with these findings where we observed a shift in bacterial community composition (Fig. 4) and difference in diversity among cropping systems (Fig. 2 and Table 2). Bacterial communities in soybean and sugarcane rhizospheres are closely clustered within each treatment while the communities in the fallowed soil are more spread-out indicating compositional homogeneity of bacterial communities within rhizosphere treatments and heterogeneity of the communities in the fallowed soil. The compositional homogeneity within each rhizosphere and the distinctiveness between them indicates the specificity of plant species to recruit microbial populations (Jaeger et al., 1999; Kourtev, Ehrenfeld & Häggblom, 2002; Costa et al., 2006; Haichar et al., 2008; Berg & Smalla, 2009). Rhizosphere bacteria are stimulated by the root exudates and the variation in the rhizodeposits from the different plant species selects for different rhizospheric communities (Garbeva, Van Veen & Van Elsas, 2004). This could explain the segregation of bacterial communities between soybean and sugarcane treatments.

The immediate effects of a soybean cover crop on below-ground bacterial community composition is shown in this study after a single year of incorporating soybean in the sugarcane monoculture system. Although this study included only three cropping systems in a single crop year, results illustrate that the use of cover crops increases bacterial diversity and impacts community composition. Results show that soybean hosts significantly higher bacterial diversity in the rhizophere than sugarcane (Fig. 2 and Table 2). As soil microbial diversity is essential in achieving crop productivity (Giller et al., 1997; Yachi & Loreau, 1999), this result suggests positive impacts of soybean as a cover crop in promoting sustainability in the long-term sugarcane monoculture system. Thus, incorporation of soybean, and perhaps other legumes as cover crops, is a viable management strategy that will likely improve the sustainability of sugarcane production systems. The potential of incorporation of leguminous plants to foster sustainability in production systems can be predicted for energycane. Both crops belong to the same genus (*Saccharum*) in which they only slightly vary on their differentially enhanced desired traits through breeding. Thus, it can be ascribed that energycanes respond to environmental influences comparably as sugarcanes do. This study also provides the groundwork for a more in-depth future assessment of the benefits of different cover crop, soil fungal communities, and different locations or soil types in sugarcane and energycane growing area.

## Conclusions

This study evaluated the immediate impact of growing a soybean cover crop on soil previously subjected to long-term sugarcane monoculture on the soil bacterial community composition. It was hypothesized that inclusion of soybean cover crop would induce changes in bacterial community diversity and structure. We found that incorporation of a soybean cover crop in long-term sugarcane monoculture fosters higher bacterial diversity and alters community composition. Soybean caters higher bacterial diversity than sugarcane and fallowed soils. Changes in bacterial community diversity and composition are driven by changes in the cropping system. We noted that these changes are influenced by crop species rather than by the soil properties measured. We also found comparable distribution of the most abundant phylotypes among cropping systems. Acidobacteria and Proteobacteria are most dominant which are consistent with their ubiquitous nature. There are few phyla which were impacted by the treatments. Association of Proteobacteria and Nitrospira to certain treatments appeared to be a response to the nutrient status of the treatments that fit to their lifestyles.

Although this study the scope of this study included only three cropping systems in a single crop year, results illustrate the immediate impact of soybean covercrop in increasing bacterial diversity and driving community composition. As microbial diversity relates to sustainability in crop production, soybean or leguminous cover crop offer a promising management strategy to offset yield decline in *Saccharum* monoculture systems. More in-depth studies are needed to quantify these effects in multiple seasons, species and soil types.

## Supplemental Information

10.7717/peerj.15754/supp-1 Supplemental Information 1 Soil chemical properties of soil samples collected from the experimental plots.

## Additional Information and Declarations

### Data Availability

The following information was supplied regarding data availability: The sequences are available at GenBank: KDPR01000001–KDPR01063647. The raw data are available in the Supplemental File.
